# Extensive comparison of salivary collection, transportation, preparation, and storage methods: a systematic review

**DOI:** 10.1186/s12903-024-03902-w

**Published:** 2024-02-02

**Authors:** Hamed Mortazavi, Amir-Ali Yousefi-Koma, Hannaneh Yousefi-Koma

**Affiliations:** 1 School of Dentistry, Shahid Beheshti University of Medical Sciences, Daneshjoo Blvd, Evin, Shahid Chamran Highway, Tehran, 1983963113 Iran; 2 Research Institute of Dental Sciences, Shahid Beheshti University of Medical Sciences, Tehran, Iran; 3 School of Medicine, Tehran University of Medical Sciences, Tehran, Iran

**Keywords:** Biomarkers, Collection, Saliva, Specimen Handling

## Abstract

**Background:**

Human saliva as a bodily fluid—similar to blood—is utilized for diagnostic purposes. Unlike blood sampling, collecting saliva is non-invasive, inexpensive, and readily accessible. There are no previously published systematic reviews regarding different collection, transportation, preparation, and storage methods for human saliva.

**Design:**

This study has been prepared and organized according to the preferred reporting items for systematic reviews and meta-analyses (PRISMA) 2020 guidelines. This systematic review has been registered at PROSPERO (Registration ID: CRD42023415384). The study question according to the PICO format was as followed: Comparison of the performance (C) of different saliva sampling, handling, transportation, and storage techniques and methods (I) assessed for analyzing stimulated or unstimulated human saliva (P and O). An electronic search was executed in Scopus, Google Scholar, and PubMed.

**Results:**

Twenty-three descriptive human clinical studies published between 1995 and 2022 were included. Eight categories of salivary features and biomarkers were investigated (i.e., salivary flow rate, total saliva quantity, total protein, cortisol, testosterone, DNA quality and quantity, pH and buffering pH). Twenty-two saliva sampling methods/devices were utilized. Passive drooling, Salivette®, and spitting were the most utilized methods. Sampling times with optimum capabilities for cortisol, iodine, and oral cancer metabolites are suggested to be 7:30 AM to 9:00 AM, 10:30 AM to 11:00 AM, and 14:00 PM to 20:00 PM, respectively. There were 6 storage methods. Centrifuging samples and storing them at -70 °C to -80 °C was the most utilized storage method. For DNA quantity and quality, analyzing samples immediately after collection without centrifuging or storage, outperformed centrifuging samples and storing them at -70 °C to -80 °C. Non-coated Salivette® was the most successful method/device for analyzing salivary flow rate.

**Conclusion:**

It is highly suggested that scientists take aid from the reported categorized outcomes, and design their study questions based on the current voids for each method/device.

## Introduction

Human saliva as a bodily fluid—similar to blood—is utilized for diagnostic purposes. However, unlike blood sampling, collecting saliva is non-invasive, inexpensive, readily accessible, and stress-free [1–4]. The exocrine contribution from each of the three major couple salivary glands (i.e., parotid saliva (PS), sublingual saliva (SLS), and submandibular saliva (SMS)) along with the saliva secreted from numerous minor salivary glands, compose the whole mouth saliva (WMS) [5, 6]. In addition, WMS contains non-exocrine components as well (e.g., micro-organisms, leukocytes, desquamated oral epithelial cells, gingival (crevicular) fluid, and the serum-like fluid derived from the epithelial mucosa) [7, 8]. In gratitude towards the contribution of the mucosal and gingival fluids, transported substances in the circulatory system are also present in the WMS [9]. Therefore, WMS meets all the requirements for its use as a diagnostic bodily fluid [10, 11]. Given the many potentials of WMS, it can replace some of the blood samplings in patients who have difficulties with blood collection (e.g., toddlers, and seniles), or in patients who have to take blood samples weekly or even daily (e.g., diabetic patients, and patients who take drugs with serious side effects such as methotrexate and warfarin) [12].

Since the end of 2019/start of 2020, the COVID-19 pandemic led to a variety of invasive and non-invasive diagnostic tests to be taken every day from millions of people [13, 14]. The COVID-19 pandemic highlighted the speed, accuracy, and feasibility of non-invasive bodily fluid sampling (e.g., saliva sampling, and collecting specimen from oropharyngeal and nasopharyngeal mucosa) for viral infection screenings in large populations [15–17].

Human saliva like any other bodily fluid utilized for diagnostic purposes, requires proper collection/sampling methods and devices, precise sampling time, appropriate handling and transportation conditions, and eventually, established storage considerations until further analysis of samples [18, 19]. The endogenous and exogenous enzymes accompanied by an unforeseeable activity and configuration are responsible for vigorous and continuous modifications of specimen [1, 18]. Moreover, contributions of different salivary glands to the composition of WMS changes in accordance to the circadian rhythms [20, 21]. Therefore, the time of sampling varies depending on the purpose of the experiment [22, 23].

Over the years, a variety of different stimulating and non-stimulating saliva sampling methods have been introduced and experimented [24–26]. Passive drooling and spitting have been the most assessed non-stimulating methods [27]. While Salivette®, Parafilm® wax and paraffin wax have been assessed as stimulating methods [28]. Some scientists believe that a non-stimulated passive drooling of saliva provides the most unmanipulated and authentic sample for further analysis [29]. On the other hand, some believe that a highly sensitive device with collective absorption abilities results in fewer redundant and inessential nano and microparticles in the samples, and consequently faster and more accurate laboratory tests [30, 31]. Nonetheless, there are still no guidelines as to whether devices are necessary for some experiments, and if necessary which devices are preferred for each test [32–34]. Moreover, the superiority or inferiority of stimulated samples compared to non-stimulated samples have not been investigated in many studies [35, 36]. From leaving samples in room temperature and analyzing them without any storage immediately after sampling, to storing samples at -80 °C for months before analysis, there are numerous handling, transportation and storage methods, each employed for different analytic purposes [37–39]. Similar to sampling methods and devices, there are no established guidelines in regards to the transportation and storage conditions of human saliva samples [40–42].

**Fig. 1:**
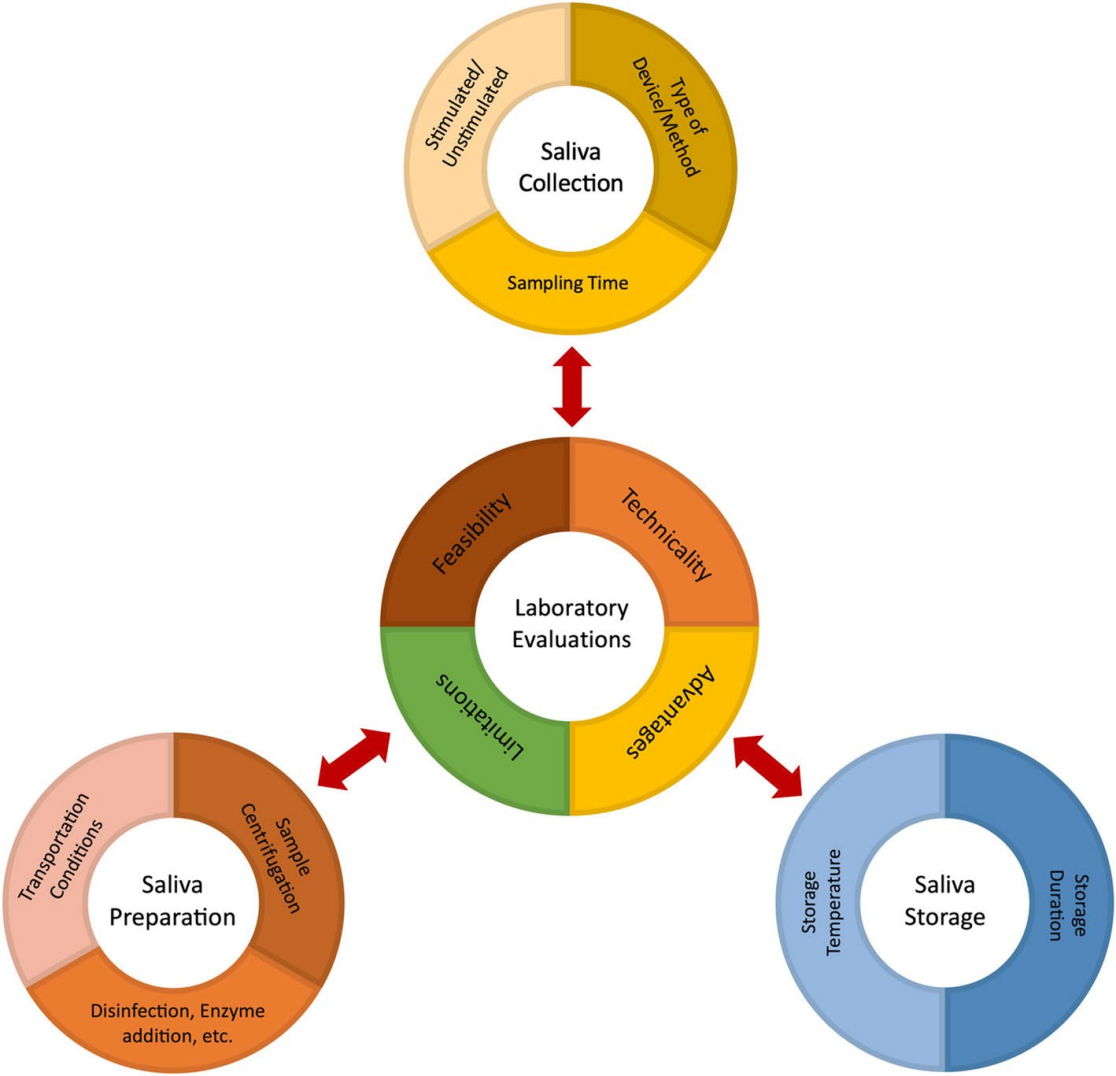
Conceptual framework of the study

Given the various diagnostic abilities of WMS and numerous features to potentially replace blood sampling in many categories of tests, WMS has gained remarkable trust as a reliable diagnostic bodily fluid [43–45]. In the past decade a special attention has been put upon creating more convenient and accurate sampling methods/devices assessed in fitting sampling times, along with proper transportation and storage conditions, depending on the tested DNA, hormone, molecule or nanoparticle [46–48]. To the best of our knowledge, there are no previously published systematic reviews on the different collection, transportation, preparation, and storage methods for human WMS in the literature, which is the main research gap of this study. The main goal for this systematic review was to gather all of the human clinical descriptive studies that have experimented different collection, transportation, preparation, and restoration techniques of human WMS. Hopefully, the extracted data reported in this review will guide clinicians and researchers in a more cohesive and accurate path in choosing the appropriate methods and devices for human WMS sampling. For a better understanding of the objectives and main purpose of this systematic review, a conceptual framework of the study has been prepared (Fig. 1).

## Materials and methods

This study has been prepared and organized according to the preferred reporting items for systematic reviews and meta-analyses (PRISMA) 2020 guidelines [49].This systematic review has been registered at PROSPERO (Registration ID: CRD42023415384). The study question according to the PICO format was as followed: Comparison of the performance (C) of different saliva sampling, handling, transportation, and storage techniques and methods (I), assessed for analyzing stimulated or unstimulated human saliva (P and O).

### Eligibility criteria

#### Types of studies

Randomized or non-randomized descriptive clinical human studies that have investigated any saliva sampling technique.

#### Population

Human participants: no exclusions regarding age, race, or gender.

#### Intervention

Collecting human saliva using stimulating or unstimulating techniques. There were no restrictions on the type of saliva (e.g., parotid saliva, submandibular saliva, and sublingual saliva). All techniques were included whether they used a specific device or not.

#### Types of outcome measures

Studies that analyzed the following outcomes were included: 1) the efficiency of the experimented stimulated and unstimulated saliva sampling techniques for each of the tested elements in the saliva (e.g., salivary flow rate, saliva DNA quality and quantity, salivary hormone levels, etc.); 2) different preparation and transportation techniques and conditions; 3) comparison of different saliva sampling times in the day; 4) patients’ preparation before and during sampling (e.g., prohibition of drinking, eating, and smoking before sampling, etc.).

### Information sources and search strategy

An electronic search was executed in Scopus, Google Scholar, and Medline via PubMed to identify eligible studies only in English language. The search was included of articles up to September 1, 2023. Search queries mentioned in Table 1 were considered for electronic search.

**Table 1:** Search queries

*Data Base*	*Date*	*Search Query*	*Results*
PubMed	September 2023	("saliva" [mesh]) AND ("sample" [mesh] OR "gather" [mesh] OR “gathering” OR "sampling" OR "collection" OR "collecting" OR "accumulation" OR "storage" OR "reserve" OR "supply" OR "stock" OR "reservoir" OR "reservation")	4859
Scopus	September 2023	TITLE-ABS-KEY (saliva) AND (sample OR gather OR gathering OR sampling OR collection OR collecting OR accumulation OR storage OR reserve OR supply OR stock OR reservoir OR reservation)	568
Google Scholar	September 2023	("saliva") AND ("sample" OR "gather" OR “gathering” OR "sampling" OR "collection" OR "collecting" OR "accumulation" OR "storage" OR "reserve" OR "supply" OR "stock" OR "reservoir" OR "reservation")	2210

### Study selection and data collection

Two reviewers (AY and HY) independently screened the titles and abstracts of articles and excluded articles based on exclusion criteria mentioned above. Selected articles were then fully read to see if they passed our inclusion criteria. In case of any disagreement a third reviewer (HM) was consulted. The data and outcomes from selected studies were then extracted and tabulated. The same reviewers performed the data extraction and any conflicts were solved by a third expert (HM).

### Data items

The collected items were as followed; (1) authors’ name; (2) year of publishment; (3) study type; (4) type of saliva; (5) sampling time; (6) number of participants; (7) participants’ gender; (8) participants’ age range and mean average age; (9) participants’ preparation before and during sampling; (10) study variables; (11) collection methods/devices; (12) sampling duration; (13) transportation conditions; (14) restoring conditions; (15) sample analysis; (16) outcomes.

### Synthesis methods

Based on the extracted data, different stimulated and unstimulated methods/techniques with or without sampling devices were widely diversified. Hence, it was not possible to perform a meta-analysis. Descriptive analysis of the data extracted from clinical studies, along with narrative and graphical synthesis was performed.

### Risk of bias assessments

The JBI Critical Appraisal Tool for risk of bias assessment in cross-sectional studies was applied for both non-randomized and randomized studies to assess their risk of bias. Two reviewers (AY and HY) independently analyzed each study using the prefabricated questions of the JBI Critical Appraisal Tool for risk of bias assessment in cross-sectional studies. In case of any dissimilarity in the results, a third reviewer (HM) was consulted.

## Results

### Study selection

**Fig. 2:**
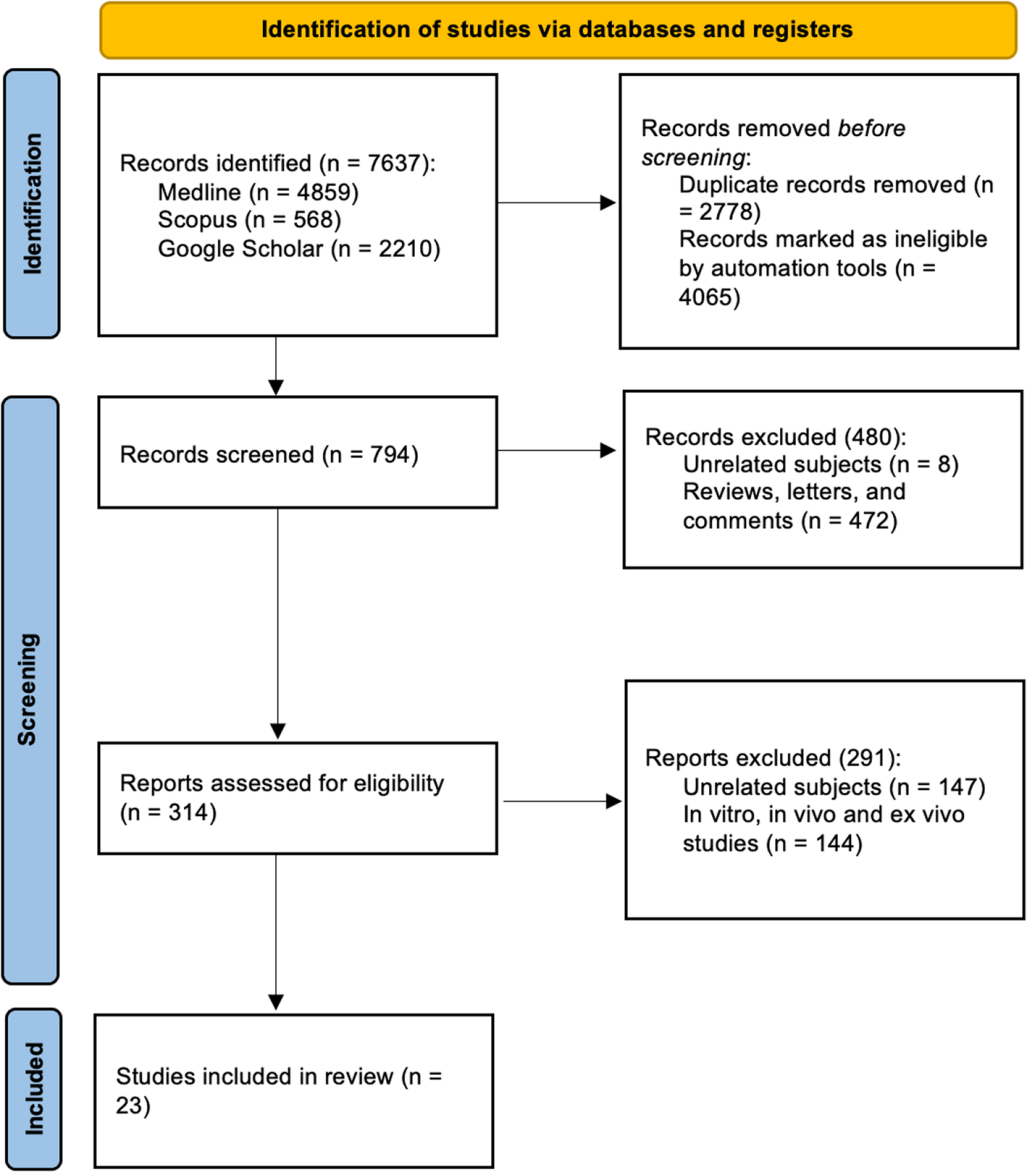
The PRISMA 2020 flow diagram of the screening and selection process

Database screening was performed and a total of 7637 articles were initially identified and 314 of them were assessed for eligibility (Fig. 2). A total of 291 studies were excluded for the following reasons; in vitro, in vivo, and ex vivo studies (*n* = 144) and unrelated subjects (*n* = 147) (Fig. 2). Hence, a total of 23 descriptive clinical human studies were included. Studies came from 13 different countries: USA (*n* = 5) [50–54], Brazil (*n* = 5) [55–59], China (*n* = 3) [60–62], Hong Kong (*n* = 1) [63], Kuwait (*n* = 1) [64], Singapore (*n* = 1) [65], Australia (*n* = 1) [66], Slovakia (*n* = 1) [67], Germany (*n* = 1) [68], Japan (*n* = 1) [69], France (*n* = 1) [70], Argentina (*n* = 1) [71], and Sweden (*n* = 1) [72]. Studies were published between 1995 and 2022: 1995 (n = 1), 2004 (*n* = 1), 2012 (*n* = 1), 2013 (*n* = 1), 2015 (*n* = 1), 2017 (*n* = 9), 2018 (*n* = 5), 2021 (*n* = 2), and 2022 (*n* = 2).

The 23 included studies were published in the following journals: Oral Diseases (*n* = 2) [57, 63], Developmental Psychobiology (*n* = 2) [52, 68], Journal of Analytical Toxicology (*n* = 2) [58, 70], Archives of Oral Biology (*n* = 2) [59, 64], Clinica Chimica Acta (*n* = 1) [65], Clinical Oral Investigations (*n* = 1) [60], Steroids (*n* = 1) [67], Clinical therapeutics (*n* = 1) [51], Scandinavian Journal of Clinical Laboratory and Investigation (*n* = 1) [55], Scientific Reports (*n* = 1) [66], Journal of Applied Oral Sciences (*n* = 1) [56], Amino Acids (*n* = 1) [69], Forensic Science International: Genetics (*n* = 1) [54], Annals of Human Biology (*n* = 1) [50], Laryngoscope (*n* = 1) [53], The Journal of Contemporary Dental Practice (*n* = 1) [72], Clinical Nutrition (*n* = 1) [62], International Journal of Environmental Research and Public Health (*n* = 1) [61], and Acta Odontológica Latinoamericana (*n* = 1) [71].

Eighteen of the included studies were funded by either public organizations or university grants [50–58, 60–62, 64, 67–71], two of the studies had no external funds for their experiments [59, 72], and three of the studies did not mention their funding/support status [63, 65, 66].

### Results of individual studies

The tabulated data of each study, their participants’ demographics, their experimented methods and their outcome are all detailed in Table 2.

**Table 2:** Different types of saliva samples, times of the day for sampling, collection techniques, collection devices, patients’ preparations, sampling duration, transporting conditions, and restoring conditions

*Author/Year*	*1) Types of Saliva,* *2) Time of the Day*	*1) Number of Patients (Health Status),* *2) Gender,* *3) Age Range or Mean Age*	*Patients’ Preparation Before and During Sampling*	*Study Variables*	*Collection Methods/Devices and Sampling Duration*	*Transportation, Sample Analysis and Restoring Conditions*	*Outcomes*
Lenander-Lumikari et al., 1995 [63]	1) WMS (stimulated) 2) At 9 AM and 11 AM	1) 16 (Healthy) 2) 5M:11F 3) Age range: 27 – 41	- All patients were instructed to eat breakfast between 7:30 and 8 AM - No eating or smoking 1 h before sampling	- Total saliva quantity - Salivary pH and buffering pH - Salivary levels of calcium, sodium, potassium, phosphate, chloride and thiocyanate	- 3 methods were assessed: A) Salivette® collection kits; with a neutral, non-covered cotton roll (chewing for 3 min) (stimulated) B) Salivette® collection kits; with a polyether roll covered with polypropylene (chewing for 3 min) (stimulated) C) Paraffin wax (melting point 46 – 48 °C); 1 g piece (control) (chewing) (stimulated), patients chewed on paraffin to softness, swallowed the produced saliva and then expectorated the secreted saliva into ice-chilled tubes for 3 min - Patients were categorized into 2 groups (8 participants each); first group used technique A at both 9 and 11 AM in the same day and both times used technique C as control immediately after A. On a separate day, they used technique B first at 9 AM and C as control immediately after B Second group had the same arrangement as group one but they used C as control before using A or B	- Salivette®: after chewing, rolls were placed in pre-weighed tubes, centrifuged at 500 g for 10 min at room temperature and then immediately pipetted into analytical tubes and frozen at − 20 °C - Paraffin wax: expired air was blown over samples, tubes were sealed with M laboratory Parafilm®. For lysozyme, albumin and lactoferrin, saliva was kept uncentrifuged. For the remaining analyses, saliva was centrifuged at 14,500 g at 4 °C for 15 min and then immediately pipetted into analytical tubes and frozen at − 20 °C	- Total average volume of WMS: 1) Salivette® (non-covered): 2.4 mL 2) Salivette® (polypropylene-covered): 1.6 mL 3) Paraffin (control): 5.4 mL - Results of pH, sodium, potassium, chloride and phosphate were similar in all 3 methods - The buffering pH was significantly lower than control in both Salivette® techniques - Calcium levels were significantly higher in Salivette® (non-covered), compared to the other 2 methods - Thiocyanate levels were significantly higher in both Salivette® techniques
Ng et al., 2004 [65]	1) WMS (unstimulated) 2) NM	1) 10 (Healthy) 2) NM 3) NM	- No eating, drinking or smoking 1 h before sampling	- Salivary DNA purity	- Each patient provided 12 mL of unstimulated sample via passive drooling	- 12 mL unstimulated samples were mixed by vortexing and inversions, then five 2 mL aliquots were dispended into 15 mL tubes and were submitted to different storage procedures: 1) S1; washing samples with PBS and extraction on the same day of sampling 2) S2; washing samples with PBS then centrifuging to produce pellet. Storing the pellet at − 70 °C for 1 week before DNA extraction 3) S3; storing samples for 1 week at 4 °C, followed by washing samples and DNA extraction 4) S4; storing samples for 1 week at 4 °C, followed by washing samples and producing pellet, then storing the pellet for 1 month at − 70 °C before DNA extraction 5) S5; storing samples for 1 month at − 70 °C, followed by washing and DNA extraction	- Purity of DNA was similar in all 5 methods (based on OD_260/280_ ratios) - All 5 methods resulted in the presence of correct sized (581 bp) single specific product - PCR bands quantification: S1 > S2 > S3 > > S4 ≅ S5
Anthonappa et al., 2012 [60]	1) WMS (unstimulated) 2) NM	1) 50 (Healthy) 2) NM 3) Age range: 5 – 10	NM	- Salivary DNA quantity - Salivary DNA purity	Each patient gave 2 mL of their WMS samples using a Oragene® self-collection DNA kit, in a single time period (spitting). The lid of the kit contains Oragene® DNA solution. After completion of sampling, WMS is immediately mixed with the solution, which stabilizes DNA and prevents bacterial growth	- 5 different restorage methods were used: 1) SC1; DNA extraction immediately after sampling 2) SC2; storing for 1 month at 37 °C before DNA extraction 3) SC3; storing for 6 months at 37 °C before DNA extraction 4) SC4; storing for 12 months at 37 °C before DNA extraction 5) SC5; storing for 18 months at 37 °C before DNA extraction	- DNA yield: SC2 ≅ SC3 ≅ SC4 ≅ SC5 > > SC1 - DNA purity (based on both OD_260/280 and_ OD_260/230_): NSD
Durdiaková et al., 2013 [67]	1) WMS (unstimulated and stimulated) 2) Between 8 to 10 AM	1) 10 (Healthy) 2) 5M:5F 3) Age range: 19 – 21	- No eating, drinking or oral hygiene procedures 30 min before sampling - No coughing during sampling (in order to avoid mucus entering the sample)	- Salivary testosterone	- All patients were asked to take all following samples with 5 min intervals; - Control; Patients dropped down their heads and let their WMS run naturally and spit it out after a while (2 mL). Control samples were not centrifuged - Repeatedly collected saliva (RS); for analyzing the effects of centrifugation immediately after sampling (the clear top-phase (100 μL) was used for ELISA): 1) RS1: centrifuged at 2,000 g for 5 min (1 mL) 2) RS2: centrifuged at 6,000 g for 5 min (1 mL) 3) RS3: centrifuged at 10,000 g for 5 min (1 mL) - Stimulated saliva (2 mL); patients were asked to touch the tip of their tongues several times with a coated cotton swab (soaked in 2% citric acid). SS samples were not centrifuged	- Following methods were used to test the salivary testosterone levels: A) Comparing the testosterone levels of unstimulated (control) and stimulated without centrifugation B) Analyzing the effect of centrifugation; comparing the results of centrifuged unstimulated samples (RS1, RS2 and RS3) against control. All of the samples were fresh and were not frozen for their ELISA assays. No processing was performed C) Analyzing the effect of different restoration temperatures and restoration times; unstimulated samples were stored in different conditions (room temperature, 4 °C, − 20 °C and − 80 °C) immediately after sampling. Samples were stored for 1 day, 1 week or 1 month. On the day of analysis, restored samples were brought to room temperature and freshly collected unstimulated was used as control	- Comparing the testosterone levels of unstimulated (control) and stimulated without centrifugation: NSD between control and stimulated - B) Analyzing the effect of centrifugation: the testosterone levels were significantly higher in control: Control > > RS1 ≅ RS2 ≅ RS3 - C) Analyzing the effect of different restoration temperatures and restoration times: Testosterone levels were not significantly different in different restoration periods (1 day, 1 week, or 1 month) in each of the restoration conditions (room temperature, 4 °C, − 20 °C and − 80 °C)
Peres et al., 2015 [51]	1) WMS (unstimulated and stimulated) 2) A) Basal and lozenge day: 9:45 AM, 10:00 AM, 10:15 AM, 10:30 AM and 10:45. AM B) Bacon day: 9:45 AM and 10:00 AM	1) 47 (Healthy) 2) 22M:25F 3) Age range: 20 – 34	- No eating or drinking 1 h before sampling - No use of antihistamines, antihypertensives, anticholinergics or diuretics - Patients with dislike of bacon or self-reported vegetarianism were excluded - Patients mouth-rinsed with water 5 min before sampling	- Salivary cortisol, - Salivary DHEA - Salivary testosterone	- The following 3 methods were used to collect patients’ saliva (2 mL from each method through a short plastic straw into a collection vial) A) An OTC anhydrous crystalline dietary supplement in lozenge form (Maxisal™) was analyzed for its saliva increasing abilities (lozenge day). One lozenge was administrated by each patient 25 min before sampling (stimulated) B) The smell of freshly baked bacon (microwaved 5 min before patients’ arrival, and left in front of patients for 5 min before sampling) was used to increase patients’ saliva flow (bacon day) (stimulated) C) Regular non-stimulated passive drooling saliva sampling (basal day) (control) (unstimulated) - For methods A and C patients provided 5 samples (2 mL each) with 15-min intervals. While for bacon method, only 2 samples were provided	- All samples were immediately frozen and stored at − 80 °C - On the day of analysis, samples were thawed and centrifuged at 1,500 g for 15 min	- Sampling duration: Lozenge > Bacon > > control - Cortisol, DHEA and testosterone concentrations: NSD amongst the 3 methods. However concentrations of all 3 hormones decreased throughout morning sessions in all 3 methods
Justino et al., 2017 [55]	1) WMS (unstimulated and stimulated) 2) Between 8 and 9 AM	1) 14 (Healthy) 2) 7M:7F 3) Mean age: 21 ± 2	- No eating, drinking or smoking 2 h and 30 min before sampling - Patients had to mouth-rinse with distilled water 5 min before sampling - The first 2 min of saliva sampling was discarded	- Salivary flow rate - Salivary total protein - Salivary nitrite -Salivary alpha amylase -Salivary antioxidant capacity	All 6 following US and SS samples were collected for all participants at the same daytime, with 5 min intervals; - Unstimulated 1) Patients accumulated saliva in their mouths for 30 s, then spat it into a tube, then 90 s of continuous spitting (US1) 2) Patients accumulated saliva in their mouths for 60 s, then spat it into a tube (US2) 3) 90 s of continuous spitting without accumulation (US3) - Stimulated: 1) Patients circulated the Salivette® cotton inside their mouth for 90 s, then the cotton was placed inside a plastic tube (SS1) 2) Patients chewed Parafilm® for 30 s, spat the accumulated saliva into tubes, followed by 90 s of continuous spitting (SS2) 3) Patients chewed mint flavored gum for 30 s, spat the accumulated saliva into tubes, followed by 90 s of continuous spitting (SS3)	All samples were immediately stored at 4 °C until all 6 sampling steps were performed for each patient, then they were centrifuged at 1976 g at 4 °C for 15 min. The supernatants were analyzed and samples were stored at − 80 °C	- Mean salivary flow rate: SS3 (3.42) > > SS1 (1.57) ≅ SS2 (1.56) ≅ US3 (1.31) > US2 (0.91) ≅ US1 (0.82) - Total protein: SS3 > > SS2 > US3 > SS1 ≅ US2 > US1 - Nitrite: SS3 > > SS2 ≅ SS1 ≅ US3 ≅ US1 > US2 - Alpha amylase: SS3 > SS1 ≅ SS2 ≅ US1 > US2 > US3 - Total antioxidant capacity: SS3 > > SS1 ≅ SS2 ≅ US1 ≅ US2 ≅ US3
Karched et al., 2017 [64]	1) WMS (stimulated) 2) NM	1) 4 (Healthy) 2) NM 3) Age range: 34 – 41	NM	- Salivary DNA quantity - Salivary bacterial quantity	Patients chewed paraffin wax and 4 mL of stimulated samples were collected	Samples (4 mL) were divided in 3 equal 1.3 mL aliquots. One tube was left on ice (WS), while the other 2 got centrifuged at 14,000 g at 4 °C for 15 min. Then the supernatant in one tube and pellet in the other tube were separated for analysis All 3 tubes were subjected to DNA purification (Masterpure™ DNA purification kit) and were preserved at − 20 °C. Samples were tested at 0 days, 7 days, 2 months, and 6 months of restoration for DNA concentration and bacterial quantities (PCR)	- DNA concentration (at all 4 time points): 1) WS ≅ pellet > > supernatant 2) There were no significant differences in different evaluation periods in each group - Mean bacterial quantities (cells/mL): 1) WS ≅ pellet > > supernatant 2) F. Nucleatum cell numbers in both WS and pellet, were significantly higher in 2 and 6 months restored samples, compared to 0 and 7 days 3) F. Alocis cell numbers in both WS and pellet, were significantly lower in 2 months of restoration compared to other time points
Lim et al., 2017 [66]	1) WMS (unstimulated)2) NM	1) 40 (Healthy) 2) NM 3) Age range: 20 – 30	- No eating or drinking 1 h before sampling - No history of drinking and drinking habits - No use of local and/or systemic antibiotics - Patients mouth-rinsed with bottled water before sampling	- Salivary DNA quantity - Salivary DNA quality - Salivary bacterial gDNA purity	- 10 of the patients (Group 1) were asked to collect their saliva using both following methods with 5-min intervals: A) Spitting into a 50 mL sterile Falcon tube B) Spitting (1 mL) into a OMNIgene™ tube (2 mL); containing 1 mL of stabilizing buffer - The remaining 30 patients (Group 2) were asked to collect their samples using all 3 following methods with 5-min intervals: A) Spitting into a 50 mL sterile Falcon tube B) Passive drooling into a 50 mL sterile Falcon tube C) Patients were asked to swish and gargle with saline solution (10 mL, 0.9% (w/v)) for 1 min before passive drooling into a 50 mL sterile Falcon tube	- Samples collected in Falcon tubes were mixed with PBS (1:1) before restoring and analysis - All of the spit and drool samples were evenly aliquoted into 1.5 mL Eppendorf tubes and stored at − 80 °C - Saline solution samples were centrifuged at 1,000 g at 4 °C for 15 min to separate cellular pellet. Then pellets were resuspended in 1 × PBS and aliquoted into 1.5 mL Eppendorf tubes and stored at − 80 °C - Variant gDNA extraction kits were assessed for all samples; Maxwell® 16 LEV blood DNA kit, in-house phenol–chloroform and QIAamp DNA Microbiome kit	1) Group 1: - NSD in quantity and quality of extracted gDNA amongst the 2 methods and NSD amongst the 3 different gDNA extraction kits 2) Group 2: - Maxwell® resulted in highest quantities of extracted gDNA. However, the data were less variable when QIAamp was used - Maxwell® showed purest qualities of gDNA (260/280 ratio) 3) Maxwell® provided the most enriched bacterial gDNA extraction (for the spit samples (50 mL Falcon tubes)) 4) there were NSD between spit, drool and saline solution regarding purity of bacterial gDNA
Roth et al., 2017 [68]	1) WMS (unstimulated) 2) 30 min after awakening, 10 min before blood draw and 20 – 30 min after the blood draw	1) 11,390 (Healthy) 2) NM 3) Age range: 3 – 4.5 month infants	- Infants with high risk genetic markers for T1D - No eating, drinking, crying or brushing teeth 30 min before sampling - No use of oral steroids 30 days prior to sampling	- Salivary cortisol	- 3 samples were collected from each patient using Salimetrics® salivary collection kit: 1) In-home sampling by parents 30 min after awakening 2) In-lab sampling by study staff 10 min before a blood draw 3) In-lab sampling by study staff 20 – 30 min after a blood draw - Each kit had 3 sorbettes (cotton pads on a stick) and a storage tube (9 sorbettes in total). All 3 sorbettes were placed under patients’ tongues (one after the other) until each cotton was saturated with saliva	- All 3 sorbettes from each sample were put in 1 storage tube. The storage tubes were centrifuges at 1,500 g for 15 min and sorbettes were discarded afterwards. Then samples were stored at − 70 °C	- Only 1.6% of samples were excluded due to insufficient quantities All samples from 3 different methods had sufficient rates of cortisol for further analysis
Garbieri et al., 2017 [56]	1) WMS (unstimulated) 2) NM	1) 20 (Healthy) 2) NM 3) Age range: ≥ 18	- No eating, drinking, kissing or smoking 30 min before sampling	- Salivary DNA quantity - Salivary DNA quality	- Passive drooling of 20 mL of US into a 50 mL polyethylene tube	- Samples were aliquoted into 1.5 mL microcentrifuge tubes - Aliquoted samples were tested immediately after sampling (T0) or were stored at − 20 °C for 3 months (T3), 6 months (T6) and 12 months (T12) until analysis - 5 different protocols were used for DNA extraction: 1) Manual purification of DNA using Oragene® DNA kit; 1 mL of saliva sample and 1 mL of suspension buffer (1:1) 2) QIAamp® DNA mini kit; with no suspension buffer 3) Samples were centrifuged for 5 min at 10,000 g, the supernatant was discarded and the pellet was resuspended in 1 mL of extraction buffer. Then 5 μL of proteinase K was added and tubes were vortexed and incubated overnight at a 56 °C water bath, samples were centrifuged again, 500 μL of 10 M ammonium was added and mixture was mixed manually for 3 to 5 min and followed by centrifuging for 15 min at 21,000 g at room temperature. Then 500 μL of its supernatant was mixed with 540 μL of cold isopropyl alcohol and were placed in refrigerator for 2 h and centrifuged for 20 min at 10,000 g at room temperature. The supernatant was discarded and 1 mL of 70% ethanol was added and tubes were centrifuged for 5 min at 10,000 g. The supernatant was discarded again and tubes were left open for 4 – 5 h. Finally DNA was hydrated in 50 μL of autoclaved deionizer water 4) InstaGene™ Matrix; 1.5 mL samples were centrifuged at 10,000 g at 4 °C for 5 min. Supernatant was discarded, 1 mL of physiological saline was added to solve the pellet, vortexed for 30 s, then centrifuged at 10,000 g at 4 °C for 5 min. 200 μL of InstaGene™ Matrix was added, vortexed for 30 s, incubated for 30 min at 56 °C, vortexed for 10 s, boiled at 100 °C for 10 min, vortexed for 10 s and finally centrifuged at 15,000 g at 4 °C for 5 min 5) InstaGene™ Matrix; similar to protocol 4 with the addition of Proteinase K and 1% SDS	1) DNA quantity: - T0: total amount of extracted DNA; protocols 4 and 5 > > protocols 1, 2 and 3 - T3: NSD between protocols 1, 3 and 5 in DNA levels - T6 and T12: NSD between protocols 1 and 4 in DNA levels - T12: protocol 5 had significantly higher levels of extracted DNA - Protocol 1: extracted DNA was efficient at all time points and the amount of DNA had NSD amongst the 3 different time points - Protocol 2: always had significantly lower amounts of DNA compared to protocol 1 at all time points - The storage time affected the DNA concentration only in protocol 3 - DNA concentration in protocol 3: T0 > > T3 > T6 > > T12 - The least amount of extracted DNA amongst all protocols and across all time points: protocol 3 at T12 2) DNA purity: - DNA purity for each protocol (protocols 1, 2, 3 and 4) were similar in different time points (T0, T3, T6 and T12). While, DNA purity in protocol 5 was rarely within the purity limits - At all time points, protocols 1 and 2 had the highest number of samples within the DNA purity limit - Number of samples within the DNA purity limit in protocol 4: T0 > T3 > T6 > T12 3) Unfragmented DNA: - T0, T3, T6 and T12: protocol 1 had 100% unfragmented DNA, which was significantly higher than protocols 2 (5%), 3 (0%), 4 (10%) and 5 (20%)
Portilho et al., 2017 [57]	1) WMS (unstimulated) 2) NM	1) 74 (32 HBV-infected and 42 healthy patients) 2) 25M:49F; 20M:12F HBV-infected and 5M:37F healthy patients 3) Mean age: 37.76 ± 11.89	- No eating or drinking, 1 h before sampling	- Salivary HBV DNA quantity	- 4 different methods were assessed from each patient on the same day: 1) Spontaneous spitting (1 – 2 mL) 2) Salivette®; the absorbent pad was placed inside the mouth for 2 min 3) Whatman FTA™ Cards; the foam tipper applicator was rubbed inside the cheek for 30 s, the applicator was pressed onto an FTA™ card until complete saturation 4) DNA-SAL™; applicator was rubbed inside the cheek for few seconds, then a small quantity of mouth rinse was swished and spat into the tube (along with the applicator)	1) Salivette®; 1 mL of PBS (pH 7.2) was added, then centrifuged at 2,000 g for 10 min 2) Whatman FTA™; cards were dried at room temperature for 1 h	HBV DNA was detected in all 4 methods. However, Salivette® had the best results
Scherer et al., 2017 [58]	1) WMS (unstimulated) 2) NM	1) 110 (Cocaine or crack-cocaine using patients) 2) 104M:6F 3) Mean age: 33.7 ± 9.4	NM	- Cocaine and crack presence in the saliva	- 2 methods were assessed for all patients: 1) MDML™ (1 mL); red lines mean positive results of drug abuse (10 ng/mL and 20 ng/mL detection cutoff). The device also stores a little bit of saliva 2) DDS2™ mobile system (0.6 mL); the collector swab is swabbed around the tongue, gums and inside cheeks (10 ng/mL and 30 ng/mL detection cutoff) - DDS2™ was assessed immediately after MDML™ with no interval	- Samples were stored at − 80 ± 2 °C - Results of the 2 tested devices/methods were compared to Liquid Chromatography-Mass Spectrometry (LC–MS)	- In comparison with LC–MS: 1) MDML™ (20 ng/mL cutoff): -Sensitivity: 100% - Specificity: 65.6% - Accuracy: 70.9% 2) MDML™ (10 ng/mL cutoff): -Sensitivity: 92.6% - Specificity: 71.1% - Accuracy: 76.6% 3) DDS2™ (30 ng/mL cutoff): -Sensitivity: 100% - Specificity: 77.77% - Accuracy: 80% 4) DDS2™ (10 ng/mL cutoff): -Sensitivity: 88.89% - Specificity: 89.15% - Accuracy: 89.09%
Ishikawa et al., 2017 [69]	1) WMS (unstimulated) 2) Between 8 AM and 12 PM (for hospitalized patients: 1.5 h (Group 1) and 3.5 h (Group 2) after breakfast and 12 h after dinner (Group 3))	1) 66 (22 oral cancer patients and 44 healthy controls) 2) 28M:38F 3) Age range: 21 – 94	- Patients mouth-rinsed with water immediately prior to sampling - For controls: no eating or drinking, 1.5 h before sampling - For all patients: no tooth-paste or rinse 1 h before sampling	- Oral cancer metabolites presence in the saliva	- Sampling for patients at home: Passive drooling (400 μL); in 50 cc Falcon tubes over 5 – 10 min - Sampling for hospitalized patients:	- Samples were immediately stored at − 80 °C until analysis - Samples were thawed and centrifuged at 9,100 g for 2.5 h, at 4 °C through a 5-kDa cutoff filter - Capillary electrophoresis time-of-flight mass spectrometry (CE-TOFMS) was assessed to quantify charged hydrophilic metabolites	- Group 3 had significantly better results than Groups 1 and 2; indicating that a 12 h fasting before sampling is ideal for quantifying charged metabolites in oral cancer patients - The salivary levels of 51 metabolites significantly differed in controls versus oral cancer patients
Cohier et al., 2017 [70]	1) WMS (unstimulated) 2) NM	1) 5 (Healthy) 2) NM 3) Age range: NM	NM	- Illicit drugs presence in the saliva	- 2 devices were assessed to identify the recent usage of illicit drugs: 1) Quantisal® (1 mL saliva and 3 mL preservation buffer); with an absorptive cellulose pad placed under the tongue until the indicator was completely blue 2) Certus® (1 mL saliva and 3 mL preservation buffer); with an absorptive polyethylene pad actively swabbed around tongue, gums and inside the cheeks until the indicator was completely blue	- Samples were stored at − 20 °C, 4 °C or room temperature for 1, 7 or 14 days	- On average the sampling duration for Quantisal® and Certus® were 3 min and 1 min respectively - At 4 °C: all drugs were stable at all time points with both devices, except for codeine, buprenorphine and methamphetamine (which their concentrations decreased at day 14) - At − 20 °C: concentrations of opiates and amphetamines decreased over the storage time with both devices - Methadone was stable and detectable at all time points and storing temperatures except for day 7
Ambers et al., 2018 [54]	1) WMS (unstimulated) 2) NM	1) 4 (Healthy) 2) NM 3) NM	NM	- Salivary flow rate - Salivary DNA quantity	MicroFLOQ® Direct swabs using the “MicroFLOQ® wet or dry traces collection procedure”	- samples were collected and diluted into 10%, 5% and 1%. Then 10 μL of all samples were pipetted onto glass microscope slides and left to dry overnight - DNA extraction was performed using the Nucleic Acid Optimizer (NAO®) Baskets and the QIAamp® kit	- MicroFLOQ® Direct swabs: the wet method had significantly better outcomes
Rosenbaum et al., 2018 [50]	1) WMS (unstimulated) 2) Prior to bed (pre-bed samples) and immediately after waking up (waking samples)	- 2005: 1) NM (Healthy) 2) NM 3) Age range: 20.9 – 22.1 - 2014: - 1) NM (Healthy) 2) NM 3) Age range: 29.6 – 31.3	NM	- Salivary cortisol - Salivary secretory immunoglobulin A (sIgA)	1) 2005: patients used 1.8 mL vials. Patients were instructed to fill about 1.5 mL of passive drooling unstimulated (all 3 samples: pre-bed, waking and 30 min after waking samples; 1 of each, 3 samples in total). Samples were kept at room temperature until they were retrieved by an interviewer in the morning 2) 2014: patients used 4.0 mL vials. Patients were instructed to fill about 3 mL of passive drooling unstimulated and repeated the sampling 1 week apart (both pre-bed and waking samples; 2 of each, 4 samples in total). Samples were kept at room temperature until they were retrieved by an interviewer in the morning	Upon the arrival of tubes in the USA, all tubes were immediately stored at − 80 °C. On the day of analysis samples were centrifuged then had their supernatants separated aliquoted into smaller tubes	- Mean sampling times: 1) 2005; 10:41 PM (pre-bed) and 6:48 AM (waking) 2) 2014; 10:24 PM (pre-bed) and 7:02 AM (waking) - Cortisol levels: samples that spent more time in the − 35 °C cooler, had significantly lower cortisol values - sIgA: samples that spent more time in the − 35 °C cooler, had significantly higher sIgA values
Mandrell et al., 2018 [52]	1) WMS (unstimulated) 2) 3 h, 2 h and 1 h before bedtime, at bedtime and 1 h after bedtime	1) 64; 39 children and 25 adolescents (Healthy) 2) 32M:32F; 18M:21F (children) and 14M:11F (adolescents) 3) Age range: 7 – 20	NM	- Salivary flow rate	- Overnight in-home salivary melatonin (DLMO) collection via passive drooling - Each patient had to collect five 100 μL samples	- Patients’ put their samples into a freezer immediately after sampling - In the morning sample tubes were returned and immediately frozen and stored at − 80 °C	- NSD in salivary properties amongst all patients in both age groups
Fakhry et al., 2018 [53]	1) WMS (unstimulated) and oral secretions 2) NM	1) 90 (with intact uterus) (Healthy) 2) 0M:90F 3) Age range: 25 – 45	NM	- Salivary immune markers	- WMS: NM - Oral secretions: a Merocel® ophthalmic sponge was placed under the tongue for 30 s then placed into a 5 mL screw cap cryovial	- Samples were first stored at 4 °C for 8 h and then at − 80 °C until the day of protein extraction - On the day of analysis, samples were thawed at room temperature for 10 min, placed in a microcentrifuge with a 0.2 μm filter, then mixed with 300 μL of extraction buffer (10 mg/mL aprotinin in PBS with 10% sodium azide). The mixture was incubated for 30 min at 4 °C, then centrifuged for 30 min at 4 °C at 14,000 rpm. Finally samples were stored at − 20 °C. Concentration of immune markers were tested by Luminex multiplex assay	- Mean concentrations of 30 out of 37 tested immune markers were significantly higher in oral secretion samples compared to WMS - Mean concentrations of IL-9, IL-33, IL-6, IL-13, TNF-α, GCSF and SCD401 were similar between the 2 methods - Oral secretions had a significantly more variable range of immune markers compared to WMS
Dos Santos et al., 2018 [59]	1) WMS (unstimulated) 2) Between 8 and 10 AM	1) 26 (Healthy) 2) 14M:12F 3) Age range: 18 – 36	- No eating, drinking or brushing teeth 1 h before sampling - Patients mouth-rinsed with water 10 min prior to sampling	- Salivary flow rate - Salivary buffering capacity - Salivary pH - Salivary total protein - Salivary enzymes activity	Passive drooling (5 mL) while patients were seated upright	- Samples were centrifuged at 10,000 g at 4 °C for 10 min - A total of 9 aliquots were made: 1) 1 aliquot was analyzed immediately after sampling without freezing 2) 4 aliquots were stored at − 20 °C 3) 4 aliquots were stored at − 80 °C - Frozen samples were stored for 3, 7, 14 and 28 days	- Salivary flow rate, buffering capacity, pH and total protein concentrations: There were NSD amongst all samples (fresh or frozen) at all time points - The activities of all enzyme were decreased in the supernatant overtime in both − 20 °C and − 80 °C stored samples. However, the activity decrease was significantly higher in − 20 °C samples - At − 20 °C: The activity of none of the enzymes were enough for analysis - At − 80 °C: Enzymatic analysis of ALT, ALP and LDH up to 3 days of storage were possible and up to 10 days of storage for TRAP and ACP
Novak et al., 2021 [72]	1) WMS (unstimulated) 2) NM	1) 52; 22 infants (under the age of 1 year) and 30 children (1 – 6 year old) (Healthy) 2) 28M:24F 3) Age range: 2 – 30 months, MA: 23 months	- No oral and maxillofacial deformities	- Total saliva quantity	- 2 methods were assessed for each patient with a 5-min interval: 1) Oral swab using Salimetrics® SalivaBio’s Children’s Swab (SCS); placed inside the mouth for 2 min 2) Salivac®; pacifier-based collection device placed inside the mouth for 2 min	NM	- Mean average amount of collected saliva: NSD; Salivac® (174 μL) > SalivaBio (158 μL)
Guo et al., 2021 [62]	1) WMS (unstimulated) 2) A) 6:00—6:30 AM; before breakfast B) 9:00 – 9:30 AM; after breakfast C) 11:00 – 11:30 AM; before lunch D) 14:00 – 14:30 PM; after lunch E) 16:30 – 17:00 PM; before dinner F) 19:00 – 19:30 PM; after dinner	1) 29 (Healthy) 2) 20M:9F 3) Mean age: 10.17 ± 1.37	- No history of thyroid diseases and intake of iodine supplements	- Salivary iodine	Passive drooling using 15 mL screw polyethylene bottles; 2 ml of US samples for each of the 6 time points	- Samples were stored at room and tested 1 week after sampling - 1 week after sampling; samples were centrifuged at 3,00 r/min for 5 min. 50 μL of saliva supernatant was mixed well with 0.95 mL 7 mmol/L ammonia water	The best sampling time for iodine analysis is after 14:00 PM
Cui et al., 2022 [61]	1) WMS, SLS, SMS and PS (unstimulated and stimulated) 2) Between 7:30 AM and 8:30 AM	- Healthy patients (control): 1) 40 2) 14M:26F 3) Mean age: 49.7 ± 3.7 - Diabetes mellitus (DM) patients: 1) 40 2) 14M:26F 3) Mean age: 50.1 ± 4.8	- Good oral hygiene on the day of sampling - No eating, drinking, smoking or oral hygiene procedures 30 min before sampling - Patients were asked to mouth-rinse with water right before sampling	- Salivary flow rate - Salivary glucose	- Saliva was collected from all 80 participants using the following methods (5 mL total sample from all 6 methods): 1) Non-stimulated whole saliva (UWS); chewing non-coated Salivette® swab for 3 min 2) Stimulated whole saliva (SWS); chewing citric-acid-coated Salivette® swab for 3 min 3) Non-stimulated sublingual/submandibular saliva (USS); putting non-coated Salivette® swab under the tongue for 3 min 4) Stimulated sublingual/submandibular saliva (SSS); putting citric-acid-coated Salivette® swab under the tongue for 3 min 5) Non-stimulated parotid saliva (UPS); placing non-coated Salivette® swab near the left parotid duct for 3 min 6) Stimulated parotid saliva (SPS); placing citric-acid-coated Salivette® swab near the left parotid duct for 3 min - At the end of all 6 methods, swabs were collected in pre-chilled polypropylene tubes placed on ice	All samples were centrifuged at − 20 °C and stored at − 20 °C	- DM patients had a significantly lower saliva flow rate than control - Stimulated samples had a significantly higher saliva flow rate than non-stimulated samples - Saliva glucose level: 1) DM patients: saliva glucose levels were significantly higher in non-stimulated samples; UPS > USS > UWS > > SPS > SSS > SWS 2) Control; NSD between different methods; USS > SSS > SPS > UPS > UWS > SWS 3) Saliva glucose levels were significantly higher in DM group compared to control in all 6 methods - In conclusion for DM patients, stimulated methods had higher saliva flow rates while non-stimulated methods had significantly higher glucose levels - The UPS (before breakfast) method, had the most correlated glucose level with blood glucose level and can serve as a non-invasive blood glucose monitoring for DM patients
Cornejo et al., 2022 [71]	1) WMS (unstimulated and stimulated) 2) NM	1) 11 (Healthy) 2) 4M:7F 3) Age range: 6 – 28 months	NM	- Salivary cariogenic streptococci count	- 2 different methods were assessed. Only 1 method was used for each patient: 1) Absorbent (unstimulated); a cotton swab was swabbed on the inner-cheek mucosa and floor of the mouth in figure of 8 motions until the cotton was completely soaked. Swabs were plated TYSCB containing Petri dishes, then placed in Eppendorf-type tubes containing PBS 2) Non-absorbent (stimulated) (1 mL); simulation was done by glove-covered fingers. Then stimulated saliva was collected from the floor of the mouth by aspiration with a plastic syringe into an Eppendorf-type tube	- Cultures were incubated at 36 ± 1 °C for 48 h - Colony forming units (CFU/mL) were analyzed	- Mean rank of CFU/mL count: Absorbent (unstimulated) (1.83) > > Non-absorbent (stimulated) (1.17) - Mean rank of counting on cultures: NSD; Absorbent (unstimulated) (1.54) ≅ Non-absorbent (stimulated) (1.46) - S. sobrinus positive results (qPCR): NSD; Non-absorbent (stimulated) (75%) > Absorbent (unstimulated) (36.4%) - S. mutans positive results (qPCR): Absorbent (unstimulated) (45.5%) > Non-absorbent (stimulated) (41.7%) - The absorbent swab method was more effective in recovering microorganisms

*Abbreviations*: *DM* diabetes mellitus, *DLMO* dim light melatonin onset, *NSD* no significant difference, *NM* not mentioned, *OTC* over-the-counter, *PS* parotid saliva, *PBS* phosphate-buffered saline, *SLS* sublingual saliva, *SMS* submandibular saliva, and *WMS* whole mouth saliva

*Note: *"≈" indicates no significant difference, ">" indicates difference between the outcomes but not significant, ">>" indicates significant difference between the outcomes

### Study characteristics

#### Study design

All of the studies were observational cross-sectional studies and none of them had any intervention on patients.

#### Demographics

Nine of the studies did not report the gender ratios of their participants. In the remaining 14 studies, 322 of the participants were females and 367 of them were males. Two of the studies did not indicate the age range or mean average age of their participants. Sixteen of the studies reported the age range of their participants and in total it ranged from 2 months to 94 years (Table 2).

#### Types of saliva

In total there were 4 kinds of investigated saliva: whole mouth saliva (WMS), parotid saliva (PS), sublingual saliva (SLS), and submandibular saliva (SMS). Each of these saliva samples were collected either stimulated or unstimulated (Table 2).

#### Sampling time

Eleven studies out of all the included studies reported their sampling times (Table 2). Only 3 of those studies compared the outcome differences of different sampling times. Sampling time varied from 6:00 AM to 20:00 PM (Table 2).

#### Patient preparations before and during sampling

Most studies asked participants to not drink, eat, or smoke 30 min to 60 min before sampling (Table 2).

#### Study variables

Studies investigated a variety of different variables in human saliva: total saliva quantity, salivary flow rate, salivary total protein, saliva pH and buffering pH, salivary minerals (e.g., calcium, potassium, iodine, etc.), salivary hormones (e.g., cortisol, testosterone, DHEA, etc.), and salivary DNA quality and quantity (Table 2).

#### Collection methods/devices

In total, 22 sampling methods/devices were assessed amongst studies (Tables 2 and 3). Fourteen of these methods/devices were used to collect unstimulated samples and the rest were used for stimulated samples (Table 3).

**Table 3:** All different stimulating and unstimulating saliva sampling methods/devices

*Method/Device*	*Description*	*Type of Saliva*		*Number of Studies Featuring The Method/Device*
		***Unstimulated***	***Stimulated***	
Passive drooling	Patients tilt their head down and let saliva accumulate in their mouth without swallowing	*		9 [53, 55, 56, 58, 59, 63, 67, 69, 72]
Spitting	Spitting can be executed either continuously or with intervals of passive drooling	*		3 [51, 60, 65]
Salivette® (non-coated)	Cotton rolls are placed inside mouth to absorb saliva	*	*	4 [50, 51, 57, 765]
Salivette® (polypropylene-covered)	Cotton rolls are placed inside mouth to absorb saliva		*	1 [50]
Salivette® (citric-acid-coated)	Cotton rolls are placed inside mouth to absorb saliva		*	1 [54]
Paraffin wax	Chewing the wax to stimulate saliva		*	2 [50, 52]
Parafilm®	Chewing the Parafilm® to stimulate saliva		*	1 [51]
Mint-flavored gum	Chewing the mint-flavored gum to stimulate saliva		*	1 [51]
Oragene® self-collection DNA kit	Similar to spitting but with a guiding tool	*		1 [54]
Cotton swab soaked in 2% citric acid	Patients are asked to touch the tip of their tongue several times with this 2% citric acid-coated cotton swab to stimulate saliva		*	1 [55]
Maxisal™ (lozenge form)	A dietary supplement to increase the secretion of saliva. Patients are asked to take one lozenge 25 min before sampling		*	1 [58]
Smell of freshly baked bacon	Patients are exposed to this smell 5 min before sampling		*	1 [58]
Salimetrics® collection kit	Each kit has 3 sorbettes (cotton pads on a stick). Each sorbette must be placed under patient’s tongue	*		2 [61, 71]
Merocel® ophthalmic sponge	The sponge is placed under the tongue for 30 s	*		1 [62]
MicroFLOQ® Direct swabs (wet or dry)	Each swab is used either dry or wet (moistened with 1μl of molecular grade water). Swabs are rubbed inside the cheeks	*		1 [64]
Whatman FTA™ Cards	A foam tipper applicator is rubbed inside the cheek for 30s	*		1 [65]
DNA-SAL™	First the applicator is rubbed inside the cheeks. Then a small quantity of mouth rinse is swished and spat into the collection tube along with the applicator	*		1 [65]
MDML™	A device used for indicating drug abuse from collecting saliva. Red line means positive result of drug abuse	*		1 [66]
DDS2™	DDS2™ is a mobile system swabbed around the tongue, gums and inside the cheeks	*		1 [66]
Quantisal®	An absorptive cellulose pad placed under the tongue until the indicator turns completely blue	*		1 [64]
Certus®	An absorptive polyethylene pad placed under the tongue until the indicator turns completely blue	*		1 [64]
Salivac®	Pacifier collection device placed inside the mouth for 2 min	*		1 [71]

*Indicating the type of saliva (i.e., unstimulated or stimulated)

#### Sampling duration

Some studies asked participants to fill a certain amount of saliva regardless of how much time it took. On the other hand some studies asked patients to use/chew on the experimented device, paraffin wax or the Parafilm® wax for a certain amount of time regardless of the total amount of collected saliva (Table 2).

#### Transportation, sample analysis and restoring conditions

Only 1 of the studies did not indicate their transportation or restoring conditions. The rest of the studies had a variety of different experimented conditions (Table 2).

### Reported outcomes

#### Sampling methods/devices

Overall, none of the 22 collection methods employed in the 23 included studies (Table 3) led to underwhelming outcomes for further laboratorial analysis. However, some of the methods outshined the rest in the studies that more than 1 method was utilized for saliva collection.

##### Salivary flow rate

In total, 8 methods/devices were assessed in this category of laboratorial tests (i.e., passive drooling, spitting, non-coated Salivette®, citric-acid-coated Salivette®, dry MicroFLOQ®, wet MicroFLOQ®, chewing mint-flavored gum, and chewing Parafilm) in 5 of the included studies [52, 54, 55, 59, 61]. Passive drooling and spitting both led to average/conventional results as unstimulated techniques. The dry MicroFLOQ® traces were the only stimulated method that had modest results. All of the 5 remaining stimulated techniques (i.e., wet MicroFLOQ®, chewing Parafilm®, chewing mint-flavored gum, citric-acid-coated Salivette®, and non-coated Salivette®) resulted in remarkable outcomes.

##### Total saliva quantity

In total, 5 methods/devices were assessed (i.e., chewing paraffin wax, non-coated Salivette®, polypropylene-coated Salivette®, Salivac®, and Salimetrics® SalivaBio®’s children’s swab) in 2 of the included studies [63, 72]. Salimetrics® SalivaBio®’s children’s swab and Salivac® were both unstimulated techniques with moderate results. Non-coated Salivette® and polypropylene-coated Salivette® were stimulated techniques with conventional results, while chewing Parafilm® wax led to remarkable outcomes.

##### Saliva pH and Salivary Buffering pH

In total, 4 methods were assessed for this category of tests (i.e., chewing paraffin wax (stimulated), passive drooling (unstimulated), polypropylene-coated Salivette® (stimulated), and non-coated Salivette® (stimulated)) in 2 of the included studies [59, 63]. Whilst chewing paraffin wax had noteworthy outcomes, the other 3 managed to lead to decent yet average laboratory results.

##### Salivary total protein

Passive drooling (unstimulated), spitting (unstimulated), non-coated Salivette® (stimulated), and chewing on Parafilm® wax (stimulated) all had respectable results [55, 59]. While chewing mint-flavored gum (stimulated) was the only method that resulted into significant outcomes [55, 59].

##### Salivary DNA quantity/concentration

Nine methods/devices were assessed in total for this variable (i.e., chewing paraffin wax (stimulated), Whatman FTA® cards (unstimulated), DNA-SAL kit (unstimulated), non-coated Salivette® (unstimulated), dry MicroFLOQ® (unstimulated), wet MicroFLOQ® (unstimulated), Oragene® self-collection kit (unstimulated), spitting (unstimulated), and passive drooling (unstimulated)) in 6 of the included studies [54, 56, 57, 60, 64, 66]. Out of these 9 methods/devices, only wet MicroFLOQ® traces led to exceptional outcomes while the rest all had conventional and accepted outcomes.

##### Salivary DNA quality/purity

All 3 methods/devices assessed in this category (i.e., spitting, passive drooling, and Oragene® self-collection kit) were unstimulated techniques and all had standard outcomes [56, 60, 65, 66].

##### Salivary cortisol

Four methods/devices were assessed for this category (i.e., smell of freshly-baked bacon (stimulated), Maxissal® (lozenge-form) (stimulated), Salimetrics® (unstimulated), and passive drooling (stimulated)). All 4 of these methods/devices had standard outcomes [50, 51, 68].

##### Salivary testosterone

Four methods/devices were assessed for this category (i.e., smell of freshly-baked bacon (stimulated), Maxissal® (lozenge-form) (stimulated), citric-acid-coated cotton swab (stimulated), and passive drooling (unstimulated)). All 4 of these methods/devices had standard and conventional outcomes [51, 67].

#### Sampling time

Only 3 out of the 23 studies had investigated the outcome differences of different sampling times during the day [51, 62, 69]. The presence of oral cancer metabolites was at its peak in samples taken between 7:30 AM and 9:00 AM [69]. Salivary cortisol, testosterone, and DHEA levels were significantly higher in samples taken between 10:30 AM and 11:00 AM [51]. Salivary iodine level was at its peak in samples taken between 14:00 PM and 20:00 PM [62].

#### Transportation, preparation, and storage conditions

**Fig. 3:**
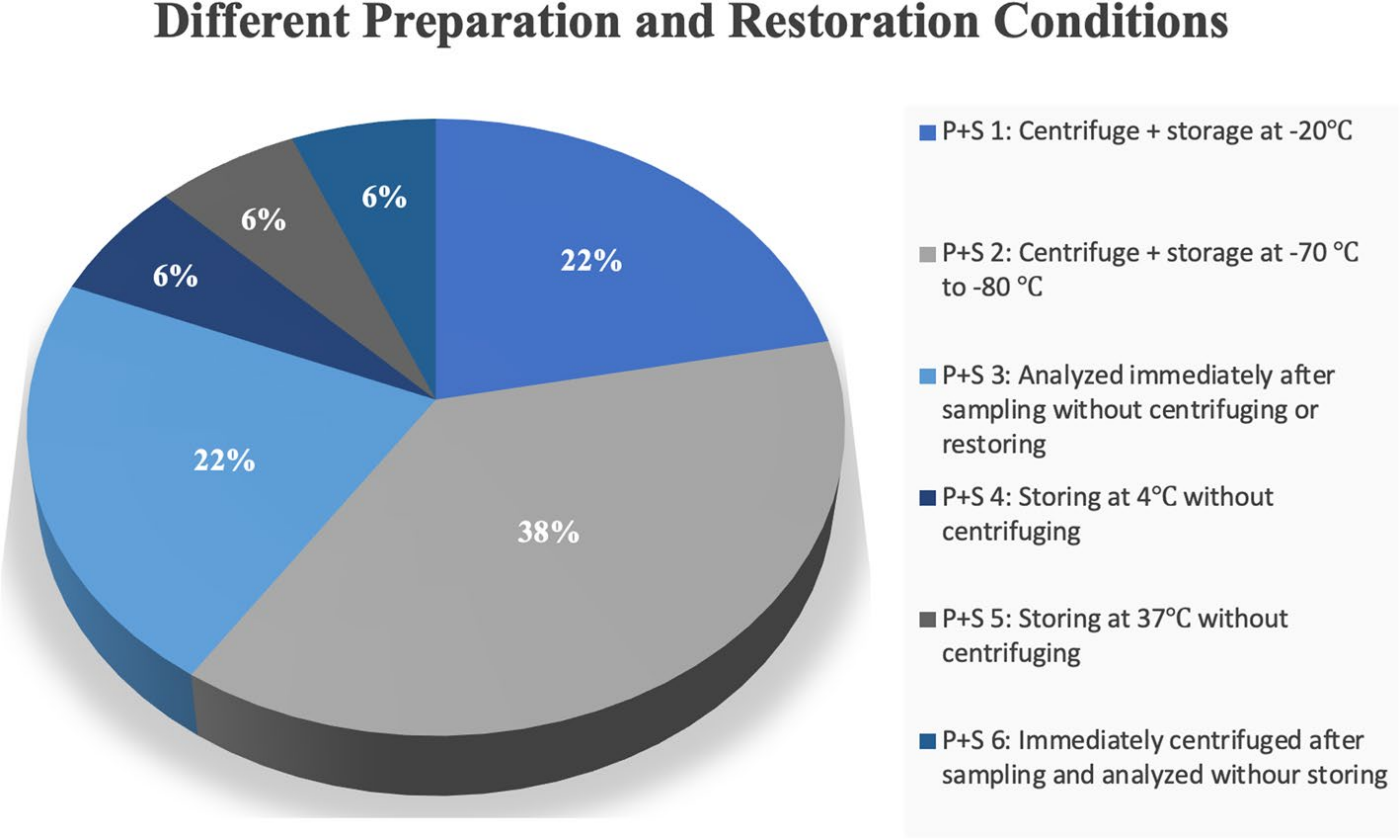
All 6 different preparation and storage (P + S) conditions, and their generality amongst included studies

All of the varied preparation and storage conditions were categorized into 6 groups. Figure 3 details all 6 methods’ descriptions (i.e., P + S 1, P + S 2, P + S 3, P + S 4, P + S 5, and P + S 6) and showcases the frequency of assessments for each method (Fig. 3). The “P + S” abbreviation used in tables and figures indicates the preparation and storage (P + S) conditions of samples before further analysis (Fig. 3, Table 4). Centrifuging samples before storing them at -70 °C to -80 °C (P + S 2) was the most assessed method (Fig. 3). Out of the 23 included studies, 5 of them compared the outcome differences of different preparation/storage methods [59, 60, 64, 65, 67]. Table 4 displays the results of all of the comparisons, along with the variables that these methods were assessed for (Table 4).

**Table 4:** Evaluative comparison amongst different preparation and storage conditions

*Study Variables*	*Comparison*	*Number of Studies Featuring The Comparison*
Salivary DNA quantity and bacterial quantity	P + S 3 ≅ P + S 1	1 [52]
Salivary DNA quality/purity	P + S 4 > > P + S 2	1 [53]
Salivary DNA quality/purity	P + S 3 > > P + S 2	1 [53]
Salivary DNA quality/purity	P + S 3 ≅ P + S 4	1 [53]
Salivary testosterone	P + S 1 ≅ P + S 2 ≅ P + S 4 ≅ P + S 6	1 [55]
Salivary DNA quantity and DNA quality/purity	P + S 5 > > P + S 3	1 [54]
Salivary enzyme activity	P + S 2 > > P + S 1	1 [69]

The 6 different preparation and storage (P + S) conditions are as followed: *P* + *S 1:* centrifuge + storage at -20°C; *P* + *S 2:* centrifuge + storage at -70°C to -80°C; *P* + *S 3:* analyzed immediately after sampling without centrifuging or storing; *P* + *S 4:* storing at 4°C without centrifuging; *P* + *S 5:* storing at 37°C without centrifuging; *P* + *S 6:* immediately centrifuged after sampling and analyzed without storing

*Note*: "≈" indicates no significant difference, ">>" indicates significant difference between the outcomes

### Risk of bias assessments

**Fig. 4:**
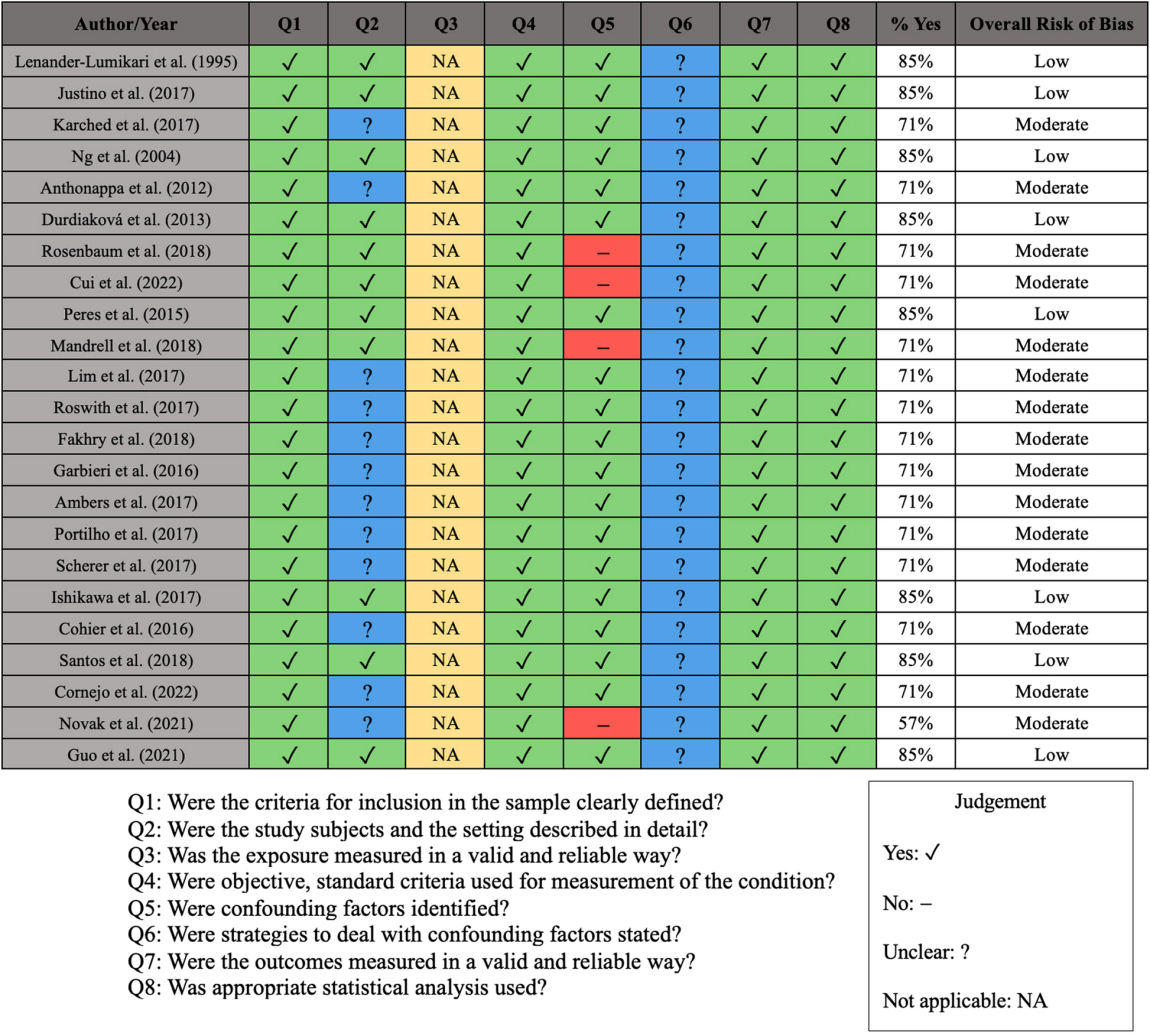
Risk of bias assessment results using the JBI Critical Appraisal Tool for risk of bias assessment in cross-sectional studies

The results of the risk of bias assessments using the JBI Critical Appraisal Tool for risk of bias assessment in cross-sectional studies are showcased in Fig. 4. Out of the 23 included studies, 8 studies had low risks of bias [51, 55, 59, 60, 62, 63, 65, 69], while the rest all had a moderate status in overall risk of bias (Fig. 4).

## Discussion

Saliva as a diagnostic bodily fluid has gained tremendous respect and trust from clinicians and scientists in regards to experiments that were only feasible through blood samplings up until couple decades ago [61, 62, 71]. Saliva is collected to analyze the oral and systematic health of patients, and has been conspicuously called *“mirror of the body’s health”* [73]*.* Saliva as an exocrine solution, intercommunicates in both intracellular and extracellular manners with the oral cavity, and is a remarkable factor in determining and ascertaining the prevalence of dental caries [74, 75]. Human WMS comprises of numerous proteins, peptides and enzymes of clinical relevance [48]. About 30% of all blood proteins are present in WMS [76]. Saliva sampling compared to blood sampling is less complicated, has a shorter sampling time, is non-invasive, and it significantly reduces costs [77–79]. There are numerous saliva sampling techniques along with varied handling, transportation, and storage methods [80, 81]. This systematic review was conducted to gather all of the clinical human descriptive studies that have investigated different collection, transportation, preparation and storage methods and techniques for WMS in different times of the day for various experiments.

Foddai et al. designed and executed a systematic review on the reliability of saliva sampling instead of blood sampling for laboratorial analysis on human autoantibodies [82]. They concluded that even though in many cases saliva sampling can be an appealing alternative to serum-based testing, standardization of the saliva sampling techniques, maintenance and detection methods must be fully investigated and addressed, which only further proves the importance and the necessity of this systematic review.

### Sampling time

WMS is commonly collected in the morning in order to have relatively equal contributions from parotid, submandibular and sublingual glands [83]. However, as mentioned before, there are various times of the day that saliva sampling could be performed depending on the type of hormone, mineral, nucleic product, or micro-/nanoparticles that are the main focus of each test [26]. For instance, if the main focus of the tests is to have high concentrations of parotid-secreted proteins (e.g., basic proline-rich proteins (bPRPs)), an early afternoon sampling is highly recommended [41]. Whilst, if scientists are mainly interested in sublingual- and submandibular-secreted proteins (e.g., salivary cystatins (type S)), then an early morning sampling is more appropriate [84, 85].

Out of the 23 included studies, only 3 of them had investigated the outcome differences amongst different sampling times (Table 2). Reported outcomes of Ishikawa et al.’s 2017 study suggest that 7:30 AM – 9:00 AM is the period of time with optimum features regarding the salivary oral cancer metabolites analyzes, while the 9:00 AM – 11:30 AM span had average results [69]. Peres et al. reported that 10:30 AM – 11:00 AM resulted into significantly higher levels of salivary cortisol, testosterone, and DHEA, while 9:00 AM – 10:30 AM showed lower levels [70]. Guo et al. disclosed that the salivary iodine is at its peak from 14:00 PM till 20:00 PM, while the 6:00 AM – 13:30 PM period had average iodine levels [62]. Since only 3 studies have reported comparative outcomes of different sampling times, and each study has focused on a different group of hormones and minerals, their reported outcomes could not be compared with each other. In order to have a comprehensive evaluation of different sampling time points/periods, there must be at least a couple of similar studies in each category of biomarkers, who have investigated the outcome differences of various sampling time points/periods. Unfortunately, that is not the case and it cannot be concluded if these reported outcomes are valid or not.

### Sampling methods and devices

Over the past four decades a variety of different stimulating (stimulated) and unstimulating (unstimulated) methods and devices have been introduced for saliva sampling [26, 48, 78, 86]. There are some on-site direct sampling techniques (e.g., SalivaDirect™) that are designed for pandemics (e.g., the COVID-19 pandemic) and other urgent situations that require collecting and analyzing numerous saliva samples from huge populations. However, our main focus in this study was methods and devices that are used by clinicians and researchers on a daily basis and not just in special and urgent occasions. Included studies utilized a total of 22 different methods (Table 3). Passive drooling, spitting, Salivette®, Salimetrics®, and chewing paraffin wax were the most assessed techniques, while the rest of the methods were only assessed in a single study.

Passive drooling is the oldest and most accessible sampling method that has been utilized as the main sampling technique for the past decades [26]. Passive drooling (n = 9) was the most utilized technique for saliva sampling in the included studies (Table 3). When assessed for salivary flow rate, pH, buffering pH, total protein, DNA quantity, DNA quality, cortisol, and testosterone, passive drooling did not show any remarkable results and was average compared to other unstimulated and stimulated methods. There was not a single category of tests where passive drooling caused significant outcomes. Even though passive drooling is still the most utilized sampling method in the literature, results suggest that stimulating techniques on general do a much better job. In a review of literature executed by Almukainzi et al. in 2022, it was suggested that passive drooling is a reliable substitute with significant amounts of accumulated WMS [87]. Even though passive drooling may not have the most desirable laboratorial outcomes compared to some stimulated sampling techniques (e.g., non-coated Salivette®), it still leads to promising results in cases where stimulated sampling techniques/devices such as Salivette® are not available.

Spitting is next to passive drooling as the most assessed method in saliva sampling in the past decades [42, 45, 88]. Spitting was utilized in a total of 3 studies [55, 57, 66] for 4 categories of outcomes (i.e., salivary flow rate, total protein, DNA quantity, and DNA quality), which led to average outcomes in all 4 of them (Table 3). Patients were asked to chew paraffin wax to stimulate saliva in 2 studies [63, 64]. Chewing paraffin wax led to significantly better results than other methods when samples were analyzed for total salivary quantity and salivary pH and buffering pH. However, chewing paraffin wax resulted in average results for DNA quantity analysis [63, 64].

Salivette® is a cylindrical cotton roll that has been assessed in both stimulated and unstimulated samplings [63, 76, 89–92]. Salivette® was the most assessed device (n = 4) amongst the included studies (Table 3). Salivette® was assessed in 3 different forms: non-coated, polypropylene-coated, and citric-acid-coated [55, 57, 61, 63]. Salivette® non-coated resulted in significantly better outcomes compared to other methods. Whilst Salivette® non-coated was only average for saliva total quantity, pH, buffering pH, total protein, and DNA quantity. Salivette® citric-acid-coated was only assessed for the analysis of salivary flow rate, and resulted into significantly better outcomes than other methods (Table 3). Salivette® polypropylene-coated was only utilized for the testing of total saliva quantity and only had average results. Salimetrics® was used in 2 studies and for 2 purposes only: saliva quantity and cortisol [68, 72] (Table 3). Salimetrics® led to average outcomes in both categories of experiments.

Overall, since the number of studies that each method was utilized for, and the categories that they were used for are vastly different and varied, a true evaluative comparison is not feasible with the current published studies.

In 2018, MacLean et al. conducted an in vivo study on the outcome differences of Salivette®, SalivaBio® Children’s swab, citric acid and passive drooling as sampling techniques for analyzing salivary oxytocin in domestic dogs [93]. They reported that SalivaBio® outperformed Salivett®, but they both had significantly better outcomes and yielded remarkably higher concentrations of oxytocin compared to passive drooling [93]. Stimulating the secretion of saliva through the taste of citric acid was also a successful method in their in vivo study [93]. Unfortunately, to the reviewer’s knowledge there is not a single descriptive human study that has tested these 4 methods in comparison with each other. However, the reported outcomes of MacLean et al. are still complied and in favor with our results that stimulating sampling techniques lead to remarkably better laboratorial outcomes.

### Handling, transportation and storage

Even though varied handling, transportation, and storage methods and techniques have been experimented in saliva sampling studies, there are still no guidelines indicating the methods with optimum outcomes [94]. All of the transportation and storage procedures assessed in the included studies of this review were categorized into 6 groups (Fig. 3 and Table 4). Reported outcomes show that centrifuging samples and storing them at -70°C to -80°C (T2) was the most assessed method (38%) (Fig. 3). Centrifuging samples and storing them at -20°C (T1) (22%), and immediately analyzing samples without centrifuging or storage (T3) (22%), were at second place in terms of assessment and utilization (Fig. 3). Storing samples at 4°C without centrifuging (T4) (6%), storing at 37°C without centrifuging (T5) (6%), and analyzing immediately after centrifuging without storage (T6) (6%), were the rest of the experimented methods (Fig. 3). A proper and evaluative comparison of all 6 of these methods, would have been feasible if all of these methods were assessed all together in a couple of single studies. However, 5 of the included studies in this review have compared some of these methods against each other [59, 60, 64, 65, 67] (Table 4). Since the compared methods, their category of utilization and their outcomes are notably varied and different, a conclusion cannot be drawn out (Table 4 and Fig. 3).

Out of the 23 included studies, only 3 of them had investigated the outcome differences of varied sampling times. And those 3 studies had experimented 3 completely different categories of salivary biomarkers. In order to have a clear conclusion on to which periods of time have the optimum capabilities for each category of salivary biomarkers, hormones, nucleic products, and minerals, a decent number of descriptive clinical human studies must be executed in the future so that their results can properly be evaluatively compared.

Only 5 of the experimented methods and devices were assessed in more than 1 study. Hence, the results of the remaining 17 methods and devices cannot be properly evaluated amongst different studies. Only 5 of the included studies had investigated the outcome differences of different sample transportation, handling, and storage techniques.

As mentioned before, one of the main challenges in the execution of this systematic review, was the lack of previously-published similar studies. Additionally, most descriptive human studies did not have their main focus on the outcome differences of different saliva sampling techniques. In general, most of the tested and investigated saliva sampling, transportation, and storage techniques and methods are relatively newly introduced to the field. Therefore, for valuable and reliable comparisons of their results, these 23 studies are simply not enough and there is a clear and urgent need for clinicians and scientists to utilize these varied methods and report their outcomes. Ideally, scientists can design and execute descriptive clinical human studies by utilizing multiple sampling, transportation, and storage techniques and methods, in order to compare their outcome differences. Doing so, a lot of the unanswered questions regarding the best saliva sampling, transportation, and storage methods and devices, can hopefully be answered. Scientists and clinicians can also investigate the outcome differences of various sampling times of the day, for each category of salivary biomarkers (e.g., minerals, hormones, nucleic acid products, glucose, etc.), different viruses, and bacteria.

## Conclusion

Passive drooling, non-coated Salivette® and spitting were the most utilized salivary collection methods/devices amongst the included studies. Non-coated Salivette®, citric-acid-coated Salivette®, and chewing paraffin wax, were the sampling methods with the most desirable outcomes in salivary flow rate, saliva total quantity, salivary pH and buffering pH, and salivary total protein. Sampling times with optimum capabilities for cortisol, iodine, and oral cancer metabolites are suggested to be 7:30 AM to 9:00 AM, 10:30 AM to 11:00 AM, and 14:00 PM to 20:00 PM, respectively. For DNA quantity and quality, analyzing samples immediately after collection without centrifuging or storage, outperformed centrifuging samples and storing them at -70 °C to -80 °C. Using non-coated Salivette® led to exceptional laboratorial outcomes for analyzing salivary flow rate. However, it is highly suggested that authors take aid from the categorized outcomes of descriptive studies reported in this systematic review and design their study questions based on the current voids for each method and device.

## Data Availability

All data generated or analyzed during this study are included in this published article.
